# Understanding Technology, Fuel, Market and Policy Drivers for New York State’s Power Sector Transformation

**DOI:** 10.3390/su13010265

**Published:** 2020-12-30

**Authors:** Mine Isik, P. Ozge Kaplan

**Affiliations:** Office of Research and Development, U.S. Environmental Protection Agency, 109 TW Alexander Dr., Durham, NC 27709, USA;

**Keywords:** power sector, CO_2_ emissions, NO_x_ emissions, SO_2_ emissions, energy use, decomposition analysis, LMDI, MARKAL, energy systems analysis

## Abstract

A thorough understanding of the drivers that affect the emission levels from electricity generation, support sound design and the implementation of further emission reduction goals are presented here. For instance, New York State has already committed a transition to 100% clean energy by 2040. This paper identifies the relationships among driving factors and the changes in emissions levels between 1990 and 2050 using the logarithmic mean divisia index analysis. The analysis relies on historical data and outputs from techno-economic-energy system modeling to elude future power sector pathways. Three scenarios, including a business-as-usual scenario and two policy scenarios, explore the changes in utility structure, efficiency, fuel type, generation, and emission factors, considering the non-fossil-based technology options and air regulations. We present retrospective and prospective analysis of carbon dioxide, sulfur dioxide, nitrogen oxide emissions for the New York State’s power sector. Based on our findings, although the intensity varies by period and emission type, in aggregate, fossil fuel mix change can be defined as the main contributor to reduce emissions. Electricity generation level variations and technical efficiency have relatively smaller impacts. We also observe that increased emissions due to nuclear phase-out will be avoided by the onshore and offshore wind with a lower fraction met by solar until 2050.

## 1. Introduction

Meeting increasing energy demand in a sustainable manner while managing air pollution has become strategically essential at a regional scale, especially for New York State, which has the third-largest economy in the United States [1]. In 2015, the New York State government committed to transforming the state’s power system by enacting climate action plans, including Clean Energy Standard (CES). CES consists of three tiers: investing in renewable energy facilities (Tier 1), preserving existing renewable power plants (Tier 2), and Zero Emission Credit (ZEC) generated with each megawatt-hour of electricity that supports “at-risk” existing nuclear facilities (Tier 3) [2]. With the contribution of three tiers, CES is designed to reduce the carbon level of energy supply by obtaining 50% of its electricity from renewable resources by 2030 [3]. Right after CES, New York State presented the “Reforming Energy Vision (REV)” plan that requires investing in energy efficiency in buildings, a clean energy economy, and regulatory reforms. Recently the state declared an even prone action by setting a target for a carbon-neutral power system by 2040 [4]. More electricity should be produced from non-fossil-based energy sources in line with the CES implementation to accomplish a transition to the cleaner power sector. This situation raises the question about how much investment should be made to which non-fossil-based electricity generation technology [3]. Non-fossil-based energy technologies’ environmental contributions should be identified to answer this question and prioritize technology options in the power sector. However, the attribution of avoided emissions resulted from the switch to non-fossil-based energy sources cannot be placed easily [5]. In the literature, primary energy equivalent (PEE) and the equal share (ES) are the most widely accepted approaches in this regard [6]. PEE and ES have both advantages and disadvantages related to their model assumptions. PEE calculates the emission savings due to the switch from fossil-based energy sources to non-fossil options under the assumption that emission gain equals the aggregate emission level of generating the same amount of electricity from fossil-based energy sources. In the PEE approach, emissions reductions are calculated based on the electricity intensity of fossil-based generation. Emission savings from a “non-fossil-based” energy source are product of the energy intensity value and the difference between the amount of non-fossil-based electricity produced in the base year and target year. Since this approach based on a single intensity value, when there is a demand reduction, PEE result may not present the sole impact of emission reduction resulting from the fuel-mix change [5].

Although there are numerous PEE applications in the literature [6,7], the main problem related to PEE is that the model necessitates two conditions to provide valid results. (1) The lion’s share of electricity generation should belong to fossil fuels; (2) the electricity demand should constantly increase between periods within the timeframe of the study. Hence, the applicability of PEE is limited for studies in which the electricity demand is stable or dropping. Under such circumstances, the ES approach can be applied to quantify the emissions reduction. ES calculates the emission changes resulting from non-fossil-based energy sources’ consumption by looking at the share of those sources within the electricity generation mix. In ES, the base year’s non-fossil-based electricity is adjusted by the total electricity output ratio in the target year to the base year. Hence, ES accounts for the emission reduction even under the circumstances in which the demand is declining [5].

However, while it is applicable to the energy systems with reducing demand, ES also has some limitations. ES approach is sensitive to the target year [5]. Hence, it is not capable of calculating the progress with respect to the targeted emission level for different years (such as 2030 and 2050 emission targets of the New York State). Another limitation that belongs to ES is that it is not able to quantify the decline in emissions due to the efficiency improvements, and fossil fuel mix changes in the system. Understanding the driving forces of emission reductions and evaluating the impact of clean energy deployment is essential for sound policy development. The index decomposition analysis (IDA) is a well-structured approach that can cope with the above-mentioned limitations of PE and ES.

Among different approaches of the IDA, owing to its advantages, including its theoretical foundation, the logarithmic mean divisia index (LMDI) is widely preferred [8]. LMDI methods provide solutions that are valid under factor test and time-reversal tests [9]. The advantages of the LMDI methodology include the adaptability of providing perfect decomposition with no unexplained residual terms, path independency, the symmetry between the decomposition ratios/differences within periods, and consistency in aggregation [10,11]. LMDI methodology also brings practical solutions due to its ease of use and result interpretation. Additionally, since non-fossil-based resources have a “zero” emission coefficient, LMDI’s ability to handle zero values [8] makes it applicable for the analysis of non-fossil-based energy sources’ contribution to emissions.

Although a large number of methods have been used to address the effect of direct and indirect factors on the emission change in the literature, LMDI is a popular methodology due to its above-mentioned features and it has been applied to different countries and regions to deal specifically with the electricity generation sector [12–14]. The LMDI methodology has become more popular as the penetration of renewable resources gets higher within the electricity generation sector [6]. Some representative studies examining the electricity sector include Ang and Goh [15] for Asia countries, De Oliveria-De Jesus [16] for Latin America and the Caribbean, Cheng et al. [17] for China, Cansino et al. [18] for Spain and Karmellos et al. [19] for the European Union countries. Despite the importance of state and regions’ carbon dioxide (CO_2_) emission mitigation actions and air emission co-benefits, comparatively less attention has been given to LMDI applications on local-scale energy dynamics [20]. Hence, only a few studies focus on state or city level emission analysis [21].

CO_2_ emissions from the power sector constitute a significant portion of global energy-related emissions. Combustion processes in the power sector are one of the major sources of sulfur dioxide (SO_2_) and nitrogen oxides (NO_x_) emissions that adversely impact human health and the environment [22]. SO_2_, which mainly comes from burning coal and oil, was found to contribute to the amplified respiratory symptoms and cause higher rates of asthma, cardiovascular diseases, premature death, impaired lung development in children [23,24]. Another primary concern with the SO_2_ is its contribution to acid rain which causes corrosion of building materials, deforestation, and acidification of waterways threatening the ecosystems. NO_x_ emissions, including NO_2_, trigger ozone formation which adversely impacts the air quality. The increased concentration of ozone in population centers leads to amplified exposure, which has been associated with an exacerbated risk for various respiratory problems (e.g., chronic bronchitis, emphysema, and asthma) [23].

Since the fuel, technology, and emission rate variables within dispatch decisions has a multi-pollutant impact, considering solely CO_2_ emission intensity can be misleading.

In the literature, there are studies that explore the multiple benefits of the fossil fuel transformation for electricity generation. They focus on air quality implications at multi-regional [25] and national scale [26,27]. Several studies have evaluated the impact of renewable-based electricity generation and its effectiveness of air emissions mitigation at local level. A large body of those studies specifically investigated the impact of renewable portfolio standards from a cost perspective considering the price elasticity of supply, emission abatement cost, and emissions perspective, including CO_2_ and other air emissions. Johnson and Novacheck [28] presented a method to evaluate the emission mitigation projections and related costs related to state-level RPS programs. They deployed a unit commitment and economic dispatch model to determine generator operations. An aggregate change for CO_2_, NO_x_, and SO_2_ for Michigan was provided from 2015 to 2030. Rather than focusing on the positive impact of renewable penetration, Vankatesh et al. [29] investigated the impact of coal power plant retirements on SO_2_, NO_X_, and related change in greenhouse gas (GHG) emissions. They use an economic dispatch model based on short-term marginal costs to examine the changes in the regional electricity generation for three regions including Pennsylvania–New Jersey–Maryland. Since they utilize a dispatch model rather than focusing on a single state, they worked on the whole grid attached to those regions. Rather than focusing on single fuel type, McDonald-Buller et al. [30] implemented a two-staged control technology investment model to explore the impact of a time-differentiated pricing system in cap and trade program for Texas and Mid-Atlantic electric power systems. They show the escalation of NO_x_ and SO_2_ emissions and the production costs under differentiated price signals. Rather than analyzing the power sector solely, Johnson et al. [31] assess the air quality and public health benefits of New York City’s commitment to reduce the greenhouse gas emissions by 80% by 2050 by also considering transportation and buildings. They deploy energy dispatch modeling for the power sector by taking each facility serving New York City into account. They also use EPA’s Community Multiscale Air Quality (CMAQ) model to estimate resulting air quality outcomes.

The main body of the existing literature deploys facility-based hourly dispatch models to analyze the transformation for CO_2_, SO_2_, and NO_x_ either focusing a specific type of generations (such as coal power plants) or an impact of one specific policy action (such as cap and trade program, CO_2_ emission target, renewable portfolio standard). The main goal of this work is to illustrate and analyze how different forces that modulate CO_2_, SO_2_, and NO_x_ emissions have evolved from 1990 to 2018 and might evolve beyond 2020 until 2050 to inform policy makers. For this analysis, we do not only explore the electricity generation but also capture the external factors together. The first-order drivers, including technology turnover, air pollution control leading to the emission rate change, fuel switching, and efficiency improvement, are considered. Besides, market- and policy-driven (second order) factors leading to emission reduction are explored.

The LMDI can only decompose the aggregate effect of the change in the electricity generated by non-fossil-based energy sources on emissions. In order to quantify the contribution of each fuel type separately, Goh and Ang [5] proposed an extension to LMDI based on the multilevel-hierarchical model presented by Xu and Ang [32]. We applied the multilevel-hierarchical LMDI approach, proposed by Goh and Ang, over the period between 1990 and 2050 to provide an overview of the driving forces on the change of emission levels from electricity generation units in the State of New York. From 1990 to 2018, reported data were used, whereas for the rest of the analysis period, results from a bottom-up techno-economic energy systems model (MARKAL-based) were included to evaluate three different future scenarios including the continuation of exiting trends, emission neutrality and emission neutrality when nuclear power plants are phase-out. The decomposition analysis has been carried out for five-year period-wise. Moreover, yearly decomposition analysis has been conducted between 2008 and 2018 to provide a detailed snapshot of the change in the power sector in the last decade.

## 2. Overview of Power Sector in New York State

**Figure 1.**
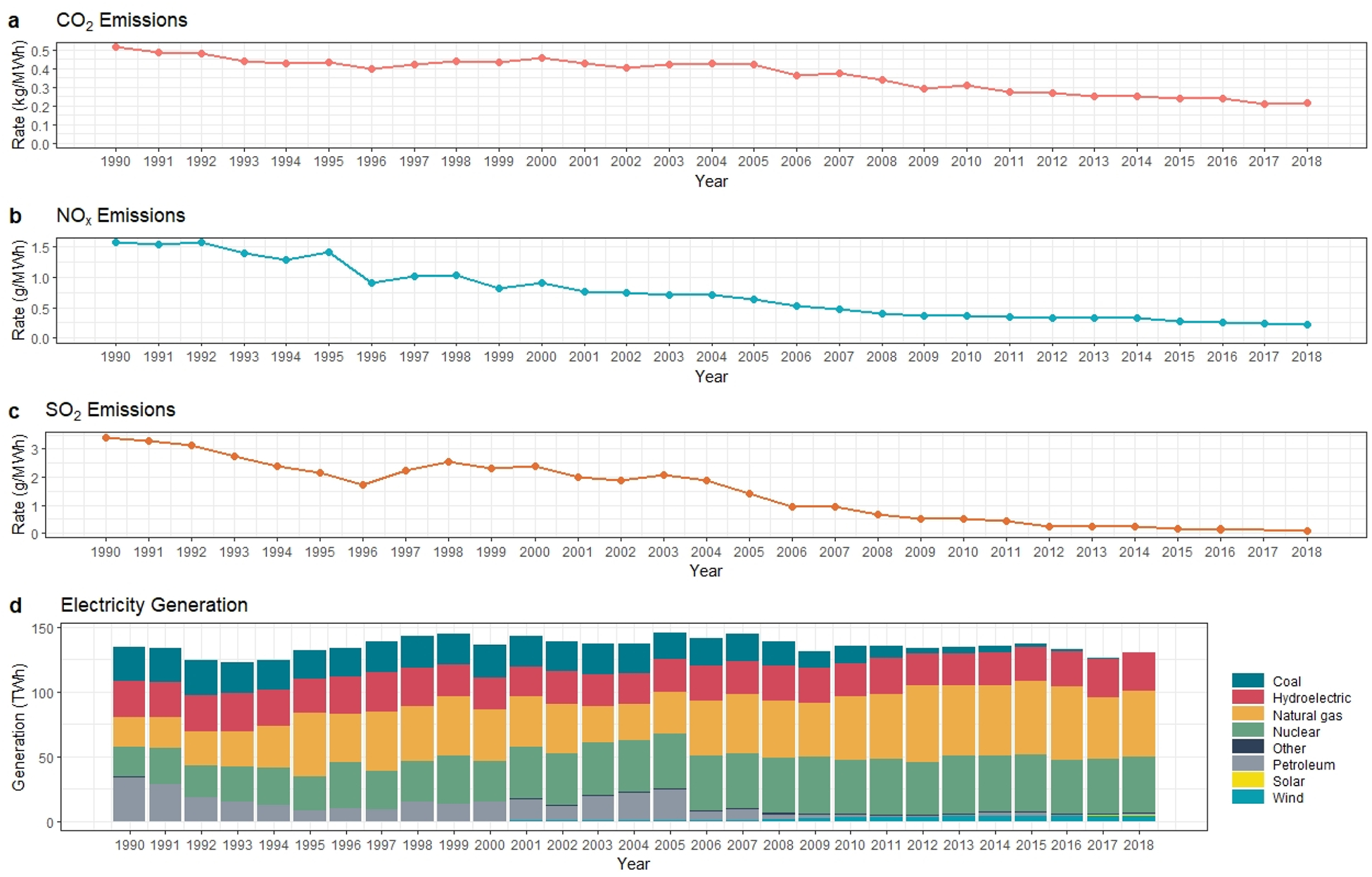
New York State electricity generation’s (**a**) CO_2_ emissions, (**b**) NO_x_ emissions, (**c**) SO_2_ emissions and (**d**) fuel consumption by primary energy source (EIA, 2019a).

The New York State electric power grid has been undergoing a transformation at an increasing pace because of the reliability and sustainability concerns. Thirty years ago, coal and oil were the major fuels generating the state’s electricity (19% and 25%, respectively) [33]. By 2018, both contributions of coal and oil to electricity generated accounted for less than 2%. Meanwhile, natural gas and renewables have increased their share in the electricity generation mix. Figure 1 illustrates the change in the electricity generation mix over time. Although nuclear power plants’ contribution remains high (accounted for about a third of the generation), the composition of the source of electricity generation has been changed over time. For instance, coal and petroleum were contributing to almost half of the electric generation mix in the 1990s, but starting in 2006, there was a shift toward natural gas.

The investments in natural gas combined cycle units have increased drastically. In 2005, natural gas was 22% of the total electricity production, whereas, in 2018, that ratio reaches approximately 40%. Figure 1 also shows the increase in the utilization of renewables with the rapid growth in the contribution of wind and solar. Renewable energy sources (hydropower, solar, and wind) provide almost 23% percent of total electricity production in the New York State in 2018.

The wholesale electricity market includes 39,295 MW total in-state electricity generation capacity including nuclear power: 5400 MW, hydropower with approximately 180 hydroelectric facilities: 5864 MW, wind:1739 MW, coal: 837 MW, dual fuel:19,112 MW, natural gas capacity: 3777 MW, oil: 2407 MW and rest of its capacity belong to other renewables [34], and imports from the New England Independent System Operator (NEISO), Ontario’s Independent Electricity System Operator (IESO), and Hydro Quebec up to 2800 MW of power [35].

The implementation of health-based air quality standards required by the Clean Air Act Amendments (CAAA) led to significant improvement in the state’s air quality [36]. The emission standards for large sources of air pollution resulting from power plants, the establishment of a cap and trade program to limit acid rain, implementation of the Montreal Protocol for ozone phase-out, and mandatory emission controls set by the U.S. Environmental Protection Agency (EPA) have transformed the electricity generation sector structure. The replacement of coal with natural gas (which has trace amounts of sulfur dioxide concentrations at levels very close to zero) and amplified renewable usage can be listed as the major changes. Air pollutant concentrations improved significantly during the analysis period.

**Figure 2.**
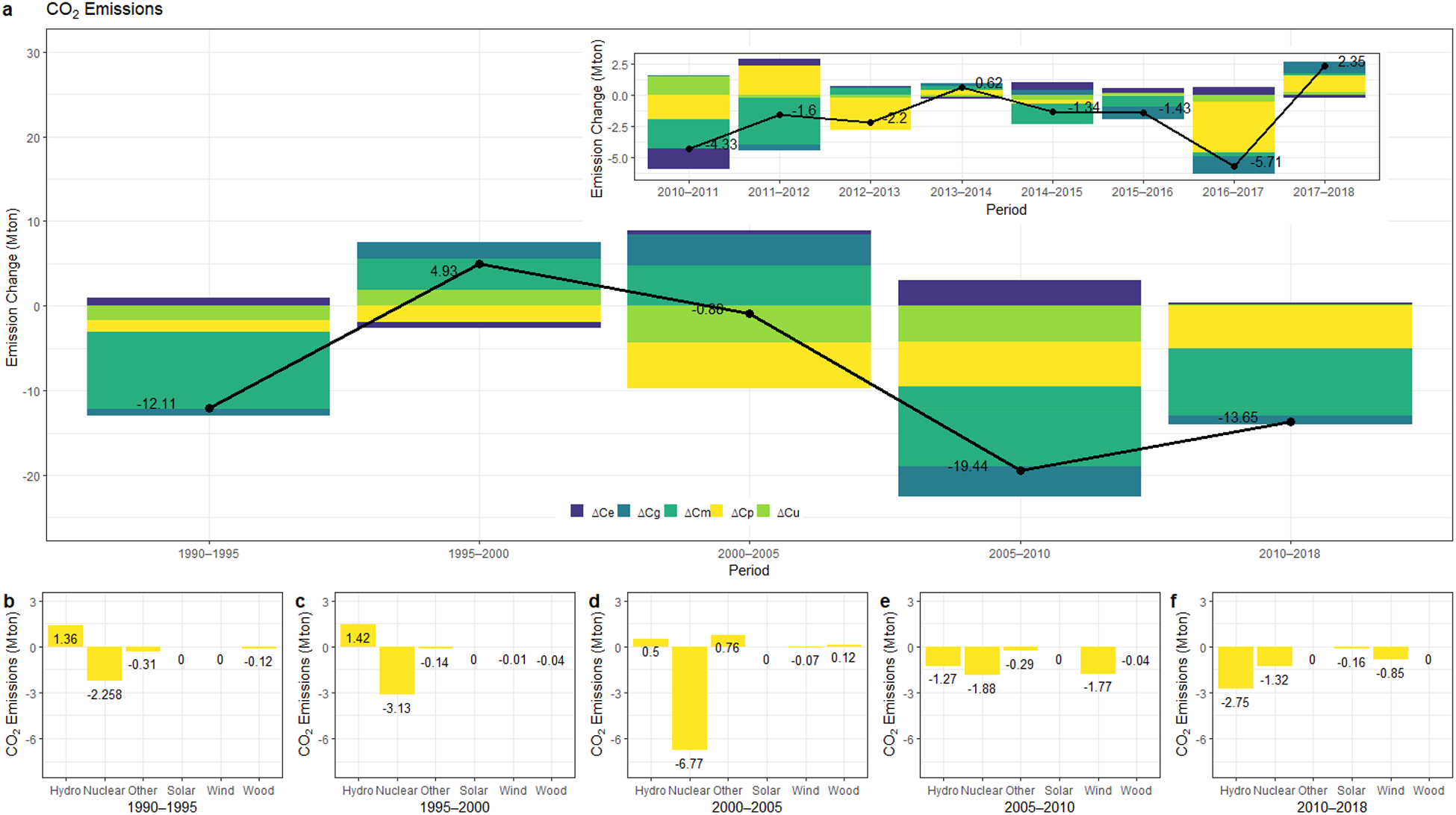
(**a**) Results of decomposition analysis for CO_2_ emissions from 1990 to 2018. Emission changes due to non-fossil-based resources for the period between (**b**) 1990–1995, (**c**) 1995–2000, (**d**) 2000–2005, (**e**) 2005–2010, (**f**) 2010–2018.

According to the State Energy Data System (SEDS), the change in the emission values that belong to the power sector is given in Figure 2 [33]. A closer look at the numbers shows that the CO_2_ emission level in 1990 was 69.09 Mton CO_2_ and decreased to 27.94 Mton CO_2_ in 2018. From 1990 through 2018, NO_x_ emissions declined by 84%. The most significant improvement was seen in SO_2_ emissions by 96% from 459.77 kt in 1990 [37].

### 2.1. LMDI Method

There exist two main decomposition analysis techniques to evaluate the factors behind the change in the carbon intensity of electricity production: structural decomposition analysis (SDA) and index decomposition analysis (IDA). SDA captures both direct and indirect effects, such as the secondary effect of a change in the demand in one sector on demand for inputs from other sectors [16]. SDA consists of detailed input-output tables. However, IDA uses only sectorial-level data which in turn has a lower data requirement. The popularity of the LMDI method within IDA is not only due to low data requirements, but it also has favorable properties from both theoretical and application viewpoints.

The LMDI method that allows decomposing changes in CO_2_ emissions into separate indexes has been preferred among the literature in various study areas including nation/economy-wide [38] and sectoral [39,40] analysis of energy consumption and related emissions. This method provides the change in driving factors to explain year-to-year differences in emissions. The preference for LMDI has also been in other study areas, such as industrial [41], logistics [42], building sectors [43]. The assessment of contributors to emissions resulting from the electricity generation sector is a field that gets a great deal of attention in the literature. Within those studies, decomposition analysis is also widely applied because of its advantages [44–46]. While mentioning the advantages from the theoretical foundation viewpoint, the LMDI methods are valid under the factor and the time-reversal tests [5]. They provide perfect decomposition with no unexplained residual terms [47]. Moreover, they can handle multiple factors in the IDA identity easily. This property is particularly relevant in studies for the electricity generation sector since four or more factors are often specified in the IDA identity. LMDI is an IDA method that is also capable of working with zero values (such as the case for renewable energy generation having zero-emission values) [48,49].

By evaluating the change in the power sector structure from the emissions point of view, a key issue is to allocate the avoided emissions from the transformation of fossil-fuel-based electricity generation into non-fuel-based electricity production, there is no standard procedure to assign avoided emissions. In this study, the LMDI additive decomposition approach is used to quantify and explain the main factors on the variation of energy-related emissions resulted from electricity generation overtime in New York State because of its theoretical properties.

The decomposition approach is formed with the breakdown of five different factors presented in Equation (1) to reveal the arithmetic change in emissions between the reference year and target year. C^t^ is the total CO_2_ emissions from electricity generation in a particular year. In the formulation and the presentation of the results, we replaced C with N and S for NO_x_ and SO_2_ emissions, respectively. ${\text{C}}_{\text{i}}^{\text{t}}$ is the CO_2_ emissions measured in a million metric ton from fossil fuel i in year t, G_t_ stands for the total electricity generation measured in megawatt-hours (MWh) for year t; Q_t_ denotes the total electricity generated (MWh) from fossil fuels for year t; Q_i_ is the total electricity output (MWh) from fossil fuel i, and F_i_ is the total energy input measured in TBtu from fossil fuel i. In the LMDI formulation p denotes the portion of the electricity generated from fossil fuels, mi is the share of the electricity generated from fossil fuel i within total electricity generation from fossil fuels whereas u_i_ represents the technical efficiency of fossil fuel i and e_i_ is the emission factor of fossil fuel i. The emission change between any given year “t” and target year “T” in emissions of CO_2_, NO_x_, and SO_2_ are subject to Equations (2)–(4), respectively for decomposition analysis. The aggregate effect could be drawn by summing the effect of electricity generation, the change in the share of fossil fuels to non-fossil based sources, the change in the share of non-fossil fuel j, changes in the fossil fuel mix, generation efficiency and the change in fuel emission factors.

(1) $$\text{C}=\underset{\text{i}}{\sum }\text{G}\frac{\text{Q}}{\text{G}}\times \frac{{\text{Q}}_{\text{i}}}{\text{Q}}\times \frac{{\text{F}}_{\text{i}}}{{\text{Q}}_{\text{i}}}\times \frac{{\text{C}}_{\text{i}}}{{\text{F}}_{\text{i}}}=\underset{\text{i}}{\sum }\text{Gp}{\text{m}}_{\text{i}}{\text{u}}_{\text{i}}{\text{e}}_{\text{i}}$$

(2) $$\Delta {\text{C}}_{\text{tot}}={\text{C}}^{\text{T}}-{\text{C}}^{\text{t}}=\Delta {\text{C}}_{\text{G}}+\Delta {\text{C}}_{\text{p}}+\Delta {\text{C}}_{\text{m}}+\Delta {\text{C}}_{\text{u}}+\Delta {\text{C}}_{\text{e}}$$

(3) $${\Delta \text{NO}}_{{\text{x}}_{\text{tot}}}={\text{NO}}_{\text{x}}^{\text{T}}-{\text{NO}}_{\text{x}}^{\text{t}}={\Delta \text{NO}}_{{\text{x}}_{\text{G}}}+{\Delta \text{NO}}_{{\text{x}}_{\text{p}}}+{\Delta \text{NO}}_{{\text{x}}_{\text{m}}}+{\Delta \text{NO}}_{{\text{x}}_{\text{u}}}+{\Delta \text{NO}}_{{\text{x}}_{\text{e}}}$$

(4) $${\Delta \text{SO}}_{2_{\text{tot}}}={\text{SO}}_{\text{x}}^{\text{T}}-{\text{SO}}_{\text{x}}^{\text{t}}={\Delta \text{SO}}_{{\text{x}}_{\text{G}}}+{\Delta \text{SO}}_{{\text{x}}_{\text{p}}}+{\Delta \text{SO}}_{{\text{x}}_{\text{m}}}+{\Delta \text{SO}}_{{\text{x}}_{\text{u}}}+{\Delta \text{SO}}_{{\text{x}}_{\text{e}}}$$

This formulation covers not only the fossil-based energy sources but also the non-fossil-based energy sources to reveal the impact of fuel share on total change. Two main types of energy sources, fossil fuels (coal, natural gas, fuel oil) that produce a high level of emissions and non-fossil fuel-based energy sources (solar, nuclear, geothermal, etc.,) are included in the model [50].

Equation (5) captures the activity effect (C_g_). ΔC_g_ represents the change in total electricity generation between period t and T informing the absolute change in emissions resulted from the variation in the total electricity output in New York State.

(5) $${\Delta \text{C}}_{\text{G}}=\sum_{\text{i}}\text{L}\left({\text{C}}_{\text{i}}^{\text{T}}{,\text{C}}_{\text{i}}^{\text{t}}\right)\text{ln}\left(\frac{{\text{G}}^{\text{T}}}{{\text{G}}^{\text{t}}}\right){\text{where}\;\text{L}}_{\left(\text{x,y}\right)}=\frac{\left(\text{x}-\text{y}\right)}{\left({\text{ln}}_{\text{x}}-{\text{ln}}_{\text{y}}\right)}\text{for}\;\text{x}\neq \text{y}$$

Equation (6) represents the fossil fuel ratio effect C_p_. ΔC_p_ measures the emission difference between two periods resulted from the change in the ratio of fossil fuel generation to total electricity generation. This effect reveals the emission change resulted from the deviation in the utilization of fossil-based energy. The fossil fuel is replaced by non-fossil-based resources as the share decreases. This parameter is also interpreted as the positive effect of the penetration of non-fossil fuel into the electricity supply mix.

(6) $${\Delta \text{C}}_{\text{p}}=\underset{\text{i}}{\sum }\text{L}\left({\text{C}}_{\text{i}}^{\text{T}}{,\text{C}}_{\text{i}}^{\text{t}}\right)\text{ln}\left(\frac{{\text{p}}^{\text{T}}}{{\text{p}}^{\text{t}}}\right)$$

We apply Equation (7) to calculate the sole effect of each non-fossil fuel-based source. ΔC_p_ is decomposed into subcategories where j represents the energy source options (j = 1 for hydroelectric, j = 2 for nuclear, j = 3 for solar, j = 4 for wind, j = 5 for other sources).

(7) $${\Delta \text{C}}_{{\text{p}}_{\text{j}}}=\sum_{\text{i}}\text{L}\left({\text{C}}_{\text{i}}^{\text{T}},{\text{C}}_{\text{i}}^{\text{t}}\right)\frac{{\text{G}}^{\text{T}}}{{\text{Q}}^{\text{T}}}\left(\frac{{\text{R}}_{\text{j}}^{\text{t}}}{{\text{G}}_{\text{j}}^{\text{t}}}-\frac{{\text{R}}_{\text{j}}^{\text{T}}}{{\text{G}}_{\text{j}}^{\text{T}}}\right)$$

The fossil fuel mix effect (C_m_) is also included as in Equation (8). ΔC_m_ measures the effect of the change within the fossil fuel mix which includes all fossil fuels (coal, natural gas, and petroleum).

(8) $${\Delta \text{C}}_{\text{m}}=\sum_{\text{i}}\text{L}\left({\text{C}}_{\text{i}}^{\text{T}}{\text{,C}}_{\text{i}}^{\text{t}}\right)\text{ln}\left(\frac{{\text{m}}_{\text{I}}^{\text{T}}}{{\text{m}}_{\text{i}}^{\text{t}}}\right)$$

Equation (9) captures the electricity generation efficiency effect C_u_. ΔC_u_ shows the change in emissions due to the change in the input to output ratio. It covers the technological enhancements of any other internal/external factors that have an impact on the input to output ratio. It represents the change in generation efficiency between any given year and target year “T.”

(9) $${\Delta \text{C}}_{\text{u}}=\sum_{\text{i}}\text{L}\left({\text{C}}_{\text{i}}^{\text{T}},{\text{C}}_{\text{i}}^{\text{t}}\right)\text{ln}\left(\frac{{\text{u}}_{\text{I}}^{\text{T}}}{{\text{u}}_{\text{i}}^{\text{t}}}\right)$$

Finally, we added the emission factor effect (C_e_) in Equation (10). ΔC_e_ captures the deviation in the emissions due to the changes in emission factors (the ratio of aggregate emissions of fossil fuel i in period t to aggregate consumption of fossil fuel i in period t). This parameter captures any external impact that is able to change the unit emission factor including air pollution control equipment and change in the chemical composition of the fuel yielding reduced emissions post-combustion.

(10) $${\Delta \text{C}}_{\text{e}}=\underset{i}{\sum }\text{L}\left({\text{C}}_{\text{i}}^{\text{T}},{\text{C}}_{\text{i}}^{\text{t}}\right)\text{ln}\left(\frac{{\text{e}}_{\text{I}}^{\text{T}}}{{\text{e}}_{\text{i}}^{\text{t}}}\right)$$

#### Data Sources for LMDI

The LMDI analysis has been carried out for the electricity generated within the New York State’s geographic boundaries. The impact of imported electricity on power sector emissions is excluded from this study. The latest U.S. Energy Information Administration (EIA) state-level detailed data on net electricity generation, which was released in January 2019, covers the 1990–2018 period. Hence, we utilized the data from 1990 to 2018 for the LMDI analysis. EIA has detailed state data including Electric Power Industry Generation by State by Type of Producer by Energy Source (EIA-906, EIA-920, and EIA-923) [37] and NYISO provides New York State Primary Consumption for Electricity Generation by Year data [34]. Wherein, the data for the proposed decomposition model is derived primarily from EIA and NYISO Power Trends Reports [51–53]. The aggregate emissions values of CO_2_, NO_x_, and SO_2_ for the New York State’s electric power industry are taken from Electric Power Industry Emissions Estimates, 1990 through 2018 [54].

### 2.2. MARKAL Model

In addition to the analysis of the historical data belonging to the New York State’s power sector, we also examined the scenarios projecting the future of the power sector emissions. The scenarios capturing future energy planning for New York State is developed through an electricity capacity expansion model built on a MARKAL framework. The MARKAL model is a mixed-integer linear programming model that solves for the least-cost system-wide solution for meeting end-use energy service demands, given the primary energy resources defined for a region by considering a wide range of available technology options in the system [55]. Researchers from academia, non-governmental organizations, and federal research laboratories have used the MARKAL framework for applications ranging from system-wide policy analysis to specific technology evaluations [56–59].

The MARKAL model typically includes: (a) End-use demands for energy services in residential, commercial, industrial, and transportation sectors (e.g., vehicle miles of travel, lumens of lighting, process heat for industrial sector); (b) supply curves in the resource sector to cover cost and emissions associated with the extraction and processing of primary energy resources such as coal, natural gas, crude oil, biomass feedstocks, and other non-biomass renewable resources; (c) energy conversion and process technologies (e.g., electricity generating units (EGUs), power plants and refineries); and (d) technologies meeting the end-use demands (e.g., LDVs, space heating, and cooling technologies, etc.,). All technologies are specified by their cost (e.g., capital, operation, and maintenance (O&M)), performance characteristics (capacity, fuel efficiency, availability), and emission rates for criteria air pollutants (CAPs) and GHGs. MARKAL provides lowest system-wide cost solution (i.e., total discounted investment, O&M, and fuel costs per technology), with the optimal mix of energy technologies and fuels, while satisfying energy balance constraints and meeting constraints on policies and regulatory standards such as air quality regulations and vehicle efficiency standards. The model calculates the resultant electricity and fuel marginal costs endogenously for all modeling years.

In this study, the demand-side representation has not been included. Hence, the electricity demanded is exogenously defined in the MARKAL model. The power sector is fully defined with all existing electricity generation capacities and available future technology options.

#### 2.2.1. Scenario Analysis

In 2018, the United Nations’ Intergovernmental Panel on Climate Change set a dead-line for greenhouse gas emissions reductions to keep the warming under the threshold value (1.5 degrees Celsius). As a response in the US, climate action plans have gained momentum within cities and states. In accordance with this momentum, New York State also launched an initiative called “Green New Deal” (GND) which consists of a set of clean energy targets to combat climate change [4]. GND aims to mandate NYS’s power sector to be carbon-free by 2040 while meeting its interim target of reaching 70% renewable electricity generation by 2030. To meet this mandate, New York State plans to double new large-scale wind and solar resources through the Clean Energy Standard while maximizing the contribution of its existing renewable resources.

To show the role of each non-fossil-based energy resource in the future of the power sector, three different scenario runs have been made. Steady State (SST), Green New Deal (GND), and Green New Deal with Nuclear Phaseout (GNDnp). SST scenario assumes the continuation of the existing trends till 2050. SST demonstrates a reference case trends in emissions while meeting the electricity demand in the system. GND simulates the transformation of the power sector under emission reduction targets. The sectoral assumptions for GND scenarios are presented in Table 1. The state is about to shut down Indian Point Nuclear Energy Center because of its aging infrastructure and resulting malfunctions. To reveal the impact of the closure of the Indian Point on the New York State’s power sector, GNDnp is set as another scenario in which no additional capacity investment in nuclear power plants is allowed. The model is only allowed to utilize the residual capacity of the existing facilities other than Indian Point in GNDnp.

In the MARKAL model, 2010 is set as the calibration year, and the analysis is conducted from 2010 to 2050. The electricity demand for 2010 and 2015 is built upon the reported data for New York State, and it is increased in line with Annual Energy Outlook 2020 Reference Case. We take the projections for electricity consumption by sectors for Middle Atlantic region till 2050 and input to the MARKAL model [60].

#### 2.2.2. Data Sources for the MARKAL

Model The data regarding NYS’s existing electricity generation capacities, including combined-cycle units are taken from numerous data resources. The status, generation capacity, and equipment characterizations of power plants are from official reports including EIA-860 [61], EIA-767 Steam-Electric Plant Operation and Design Report [62], National Electric Energy Data System [63]. To represent the state’s power sector, the primary energy supply potentials, including a reserve for fossil fuels, potential capacity, and annual limits for renewables are also exogenously defined in the MARKAL model. For future electricity generation units, a detailed technology database is prepared with possible efficiency and cost improvements for each alternative source of energy and included in the MARKAL model. The model is used to illustrate how the electricity supply is realized under alternative scenarios and how emissions and system cost change with respect to those scenarios. For detailed information including the formulations, model assumptions, and data sources, please see the model documentation [60].

## 3. Results

### 3.1. The Analysis of CO_2_ Emissions from 1990 to 2018

Between 1990 and 2018, there were sharp fluctuations in CO_2_ emission values, but the data showing positive changes were less than the data showing negative deviation in CO_2_ rates. From 1990 to 2018, New York State’s power sector CO_2_ emissions dropped by 60% and decreased to 27.9 Mton CO_2_ in 2018. During the analysis period, the major components that contributed to the total emission change were C_m_ and C_p_ as deduced from Figure 2a.

The fossil fuel mix change (ΔC_m_) had a significant impact on the decrease in CO_2_ emissions considering the whole modeling period. The share of coal within fossil fuel-based energy sources substantially decreased by a large margin to 3% from 31% in the electricity generation sector. For the same period, the share of natural gas increased to 94% within the fossil-based electricity generation. Especially after 2005, numerous factors amplified the natural gas-fired electricity generation. Ease in natural gas delivery with the expansion of pipelines and the upswing of the coal prices, a decline in the cost of natural gas due to the increase in the domestic shale gas formations were some of the major market-driven factors [64]. As a result, the production of natural gas reached a peak level in 2011 and decreased the price of natural gas drastically from 9.65 $/thousand cubic feet in 2008 to 4.18 $/thousand cubic feet in 2018 in the US [65]. Accordingly, the consumption shares of fossil fuels with comparatively low carbon content increased over the modeling period. The impact of the availability and the market price of natural gas on the electricity generation mix can be seen explicitly by looking at ΔC_m_. Specifically, in the 2005–2010 and 2010–2018 periods, this change in the fossil fuel-based electricity generation mix resulted in the highest decline in the emissions level, and it accounted for 9.45 Mton CO_2_ and 7.89 Mton CO_2_ emission reduction respectively.

ΔC_p_ represents the deviation in emissions relative to the changes in the share of fossil fuels in the electricity generation mix. The second most substantial decrease in carbon emissions was due to C_p_ for New York State case over the entire period of 1990–2018 which was equivalent to 16 Mton CO_2._. C_p_ reached its highest value with 5.5 Mton CO_2_ and 5.25 Mton CO_2_ emission reduction, respectively in the 2000–2005 and 2005–2010. Although C_p_ led to a decrease of 3.24 Mton CO_2_ between the 1990–2000 period, this value reached its peak level with 4.26 Mton CO_2_ from 2008 to 2009 after New York adopted its first RPS in 2004.

The third main component of the emission mitigation in the power sector is C_u_ which presents the emission reduction attributed to the change in efficiency. During the analysis period, we observe an upswing and downswing phase of emission change because of the efficiency effect. However, the cumulative effect of C_u_ from 1990 to 2018 was negative. The efficiency changes reduced 8.63 Mton CO_2_ emissions, which accounted for 21% of the total emission reduction. While interpreting the gain from C_u_, we should keep in mind that New York State has one of the oldest electricity generation infrastructures. Nationwide, the capacity-weighted average age of power plants is 29 years, whereas New York’s power plant fleet has an average age of 36 years [51]. The newly added, lower- or zero-emitting capacity in the New York electricity generation system equals 11,846 MW, and nearly 7000 MW of higher-emitting capacity has retired or suspended since 2000. This structural change resulted in more efficient electricity generation [52]. Thus, the total efficiency gains mainly resulted from the retired or suspended coal power plants plus the natural gas combined cycle capacity expansion.

C_g_ represents the activity change effect. During the study period, the aggregate effect of C_g_ was positive. However, from 1990 (136 TWh) through 1995 (133 TWh) and 2005 (147 TWh) to 2010 (137 TWh), there were decreasing trends as presented in Figure 2a. These declines in electricity generation resulted in emission reduction during those periods. Between 2005 and 2010, a negative deviation in electricity generated because of the economic turndown resulted in approximately 3.50 Mton CO_2_ reduction (7% of total reduction). Overall, the effect of C_g_ was not a significant determinant of emission change for the New York State case. Similarly, C_e_ was not a significant contributor.

Figure 2 also depicts the positive effect of RPS for the period of 1990–2018 in terms of CO_2._ The change in emissions resulted from the positive impact of renewables, accounted for more than 5 Mton CO_2_ from 2005 through 2010, whereas this value was equal to 5.08 Mton CO_2_ between 2010 and 2018. Exploring the differences between the effects of non-fossil-based resources helps reveal how different clean energy types contribute to emission reductions for each pollutant analyzed. Hence, the extension of LMDI through Equation (7) decomposes the sole effect of each non-fossil resource in electricity generation. Figure 2b–f depicts the resource-based emission reduction contributions of non-fossil-based energy options from 1990 to 2018, including the environmental benefits of using non-fossil-based energy resources.

Electricity generation from hydropower plants varies from year to year mainly due to water availability during the analysis period. The impact of this fluctuation on CO_2_ emissions is presented in Figure 2b–f. In the periods 1990–1995, 1995–2000, and 2000–2005 there was a significant decline in electricity generation from hydropower plants. Accordingly, we observe an increase in CO_2_ emissions (1.36 Mton CO_2_, 1.42, Mton CO_2_, and 0.50 Mton CO_2_ respectively). Although there exist 4.02 Mton CO_2_ reductions from 2005 to 2018, the net effect of hydropower plants was limited to 0.74 Mton CO_2_ reductions from 1990 to 2018.

For New York State, nuclear power has long been the largest and reliable, clean source of electricity. From 1990 through 2018, nuclear was the leading contributor to the decrease in emission levels within the non-fossil-based energy sources. U.S. EPA established the Acid Rain Program in 1995 under the CAA Amendments to limit NO_x_ and SO_2_ emissions from power plants, which resulted in the reduction of the electricity mainly generated from coal [66]. Hence, starting in 1995, we observe a sharp increase in electricity generation from nuclear power (35.2 TWh) starting until 2001 (40.4 TWh). Nuclear energy approximately produced 32% of New York State’s total electricity generation. It counted more than 50% of the state’s carbon-free power. Within the 1990–2018 timeframe, emission gain, which belonged to nuclear power plants, was 15.36 Mton CO_2_. However, for the period between 2013 and 2014, 1.13 Mton CO_2_ was emitted due to the reduced electricity production by nuclear facilities.

Regarding solar, although New York operates over 58,000 residential PV systems (Kaatz, 2017) and behind-the-meter solar will continue to expand in New York, in our study the solar only considers the effect of the “utility-scale.” Electricity generation from rooftop solar PV is not considered. Between 2010 and 2018, the total CO_2_ contribution that could be attributed to utility-scale solar power was 0.16 Mton CO_2_. Despite New York State’s high solar potential, the contribution remained limited during the study period due to its scale. Although solar did not contribute significantly to the emission reduction compared to other available renewable options, the wind showed a promising progression. Wind accounted for only 0.01% in 2000, whereas in 2015 it was equal to 2.6% of total generation. The total emission gain was 2.70 Mton CO_2_ until 2018.

### 3.2. The Analysis of NO_x_ and SO_2_ Emissions from 1990 to 2018

**Figure 3.**
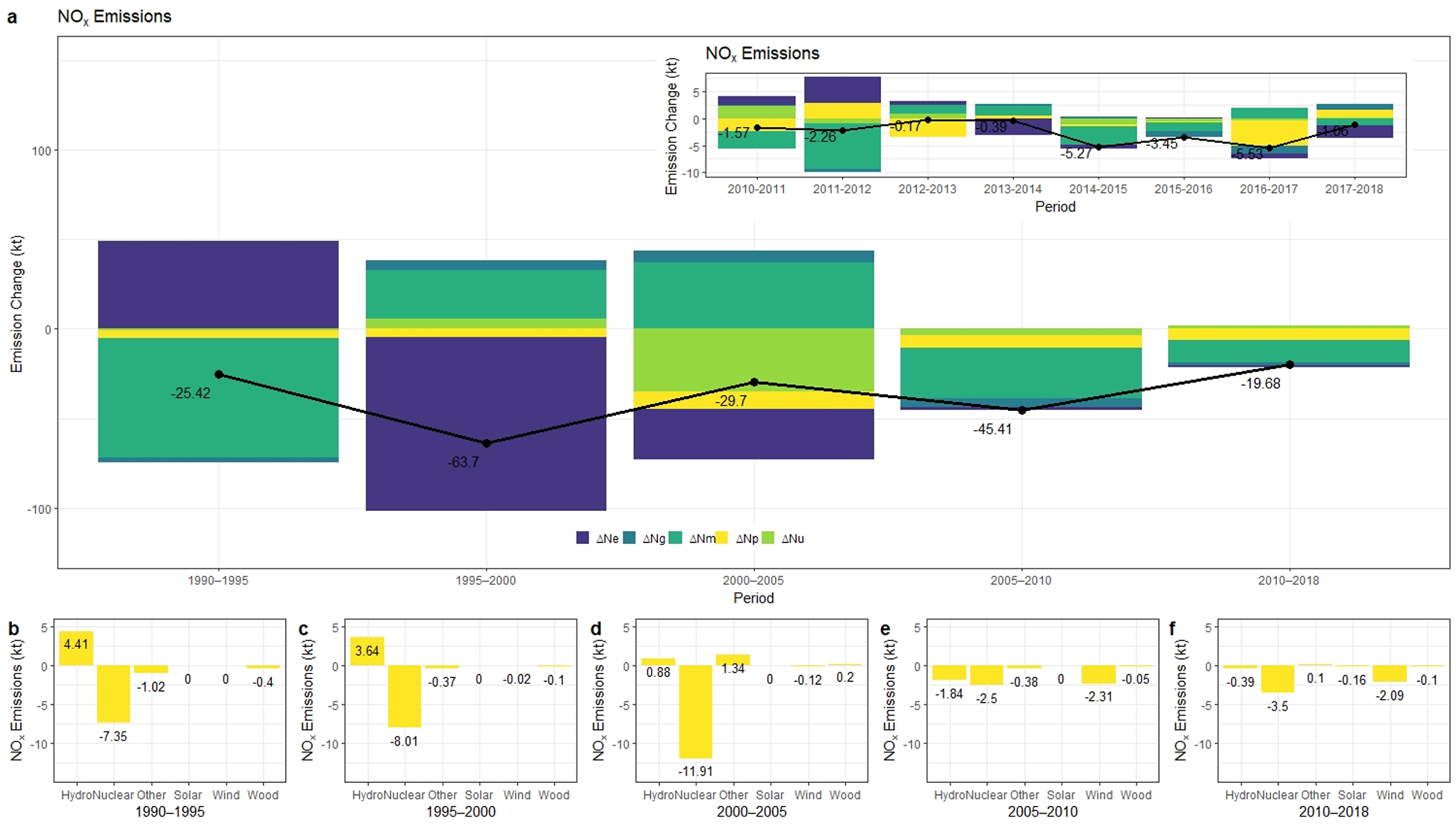
(**a**) Results of decomposition analysis for NO_x_ emissions from 1990 to 2018. Emission changes due to non-fossil-based resources for the period between (**b**) 1990–1995, (**c**) 1995–2000, (**d**) 2000–2005, (**e**) 2005–2010, (**f**) 2010–2018.

**Figure 4.**
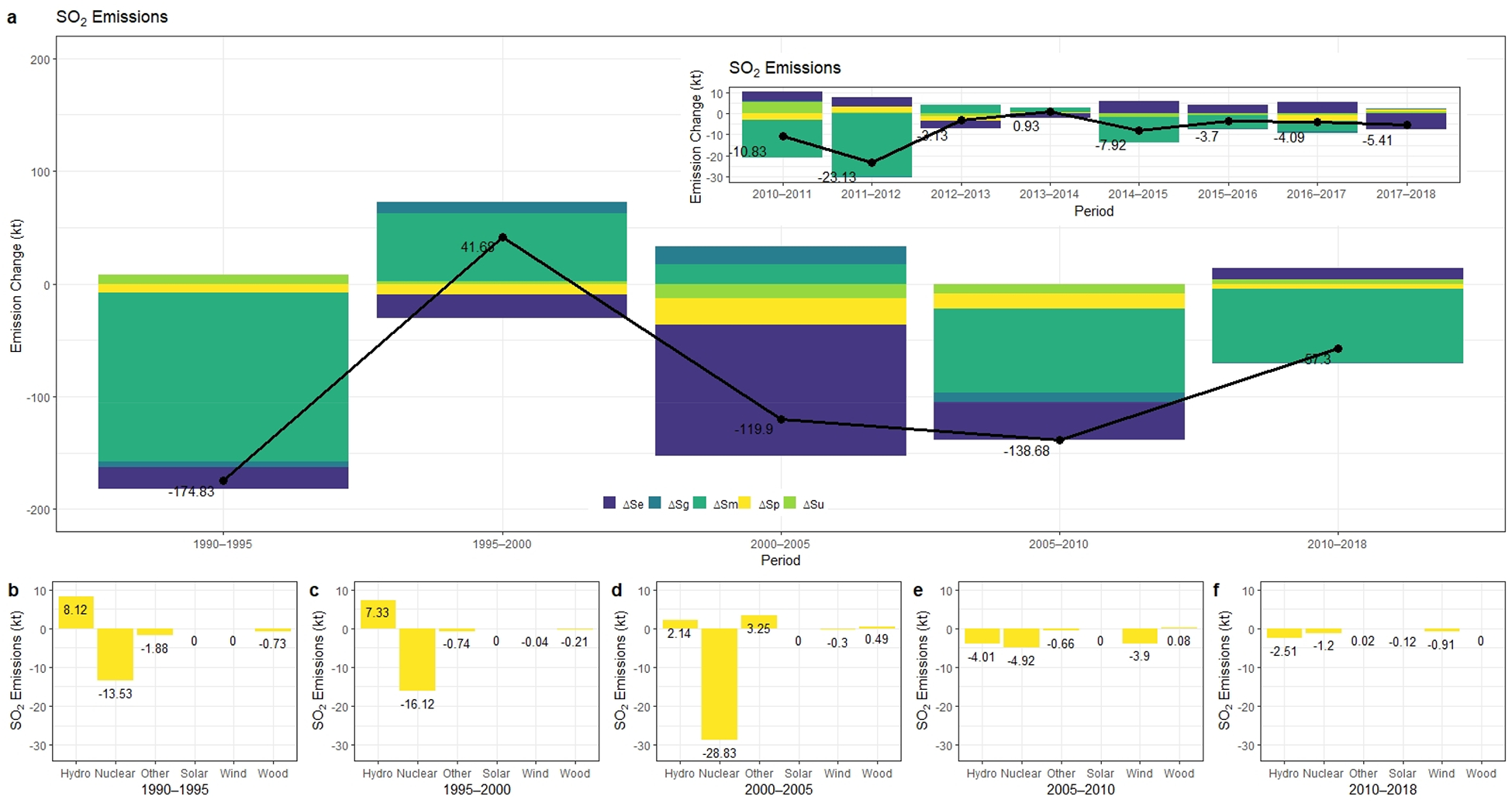
(**a**) Results of decomposition analysis for SO_2_ emissions from 1990 to 2018. Emission changes due to non-fossil-based resources for the period between (**b**) 1990–1995, (**c**) 1995–2000, (**d**) 2000–2005, (**e**) 2005–2010, (**f**) 2010–2018.

In this study, we also conducted an LMDI analysis on NO_x_ and SO_2_ emissions. The change in those emissions are presented in Figures 3 and 4, respectively. According to annual emission trends, NO_x_ emissions consistently decreased and fallen to 28.89 kt in 2018. The total NO_x_ emissions of power plants dropped 86% from 1990 through 2018, whereas SO_2_ emitted by power plants declined by 98% and reduced to 10.74 kt in 2018. Although SO_2_ emissions had decreased substantially throughout the modeling period, there was an increase from 2000 to 2005, as given in Figure 4a.

For New York State power sector, the change in the emission factor ΔN_e_ and the effect of changes in fossil fuel mix ΔN_m_ were the primary driving forces contributing to the downswing in NO_x_ emissions from 1990 to 2018. ΔN_m_, covering the impact of fuel switching toward lower sulfur fuels such as natural gas, illustrates also the magnitude of emission change due to the effect of Clean Air Act Amendments after 1990. The reason for this decline was two-fold. First, natural gas was cleaner-burning fuel than coal and petroleum, and second, natural gas combined cycle plants were typically more efficient than coal-fired power plants. Especially for the period between 1990 and 2005, we observe that the change in fuel emission factor effect was the most important driver of the NO_x_ change. 76.29 kt reduction was due to the change in the emission factor, whereas after 2005 this value of change was equal to 3.27 kt. From 2005 to 2018, it can be seen that fuel mix change is the most important factor within other drivers as given in Figure 2a.

Between 1990 and 2018, the effect of fossil intensity changes in the generation mix accounted for 17% of the reduction whereas in the last decade higher penetration of renewables amplified this ratio approximately to 30%. The changes in total electricity generation played a minor role in decreasing co-pollutant emissions (less than 3% reduction in NO_x_ and SO_2_).

Turning to changes in SO_2_ emissions from New York State power sector, there was a sharp decline for the 1990–2018 period. The emission rate of SO_2_ that belongs to the power plants in New York State went down under 0.3 lbs/MWh from 6.8 lbs/MWh. However, the degree of electricity generation, the effect of the change in the share of fossil fuels to non-fossil-based sources, the effect of changes in the fossil fuel mix, and generation efficiency effects on emissions differ by period. We found that the highest reduction in SO_2_ was mainly due to “the effect of changes in fossil fuel mix.” ΔS_m_ also represents the switch in the fuel mix toward lower sulfur content. Over the modeling period, aggregate reduction in SO_2_ emissions which was driven by ΔS_m_ was equal to 212.45 kt which accounted for 47% of the negative change.

Furthermore, between 2005 and 2018, ΔS_m_ was even more influential as 72% of the reduction was attributed to the change in the share within the fossil fuel consumption. ΔS_e_ also played an important role in inhibiting SO_2_ emissions. 179.90 kt of SO_2_ was driven by the change of emission level per unit fuel consumption whereas ΔS_p_ and ΔS_u_ affected the 59.35 kt decrease and the 8.65 kt decrease respectively as given in Figure 4a. Throughout the modeling period, the effect of generation efficiency, the effect of the change in the share of fossil fuels to non-fossil-based sources, and the change in total electricity generation had less influence on SO_2_ emission changes. From 1990 through 2005, the desulfurization of electricity generation mainly resulted from the change in emissions factor, which was due to the declining sulfur content of the coal and the increased installation of flue gas desulfurization systems (FGD). On the other hand, after 2000, most FGD-based reduction opportunities had already been implemented, the shift from coal to other non-fossil fuel-based electricity generation mainly drove additional emission reductions of SO_2_ for New York State.

Results show that the sulfur content and heat content of the coal also improved over time, plus coal power plants started installing FGDs, which helped lowering unit SO_2_ emission levels significantly. From 2010–2018 the change in the share of fossil fuels in fossil fuel-based electricity generation was still an essential factor in the negative deviation rather than non-fossil-based electricity generation effect in terms of SO_2_ emissions.

### 3.3. The Analysis of the Emission Scenarios

**Figure 5.**
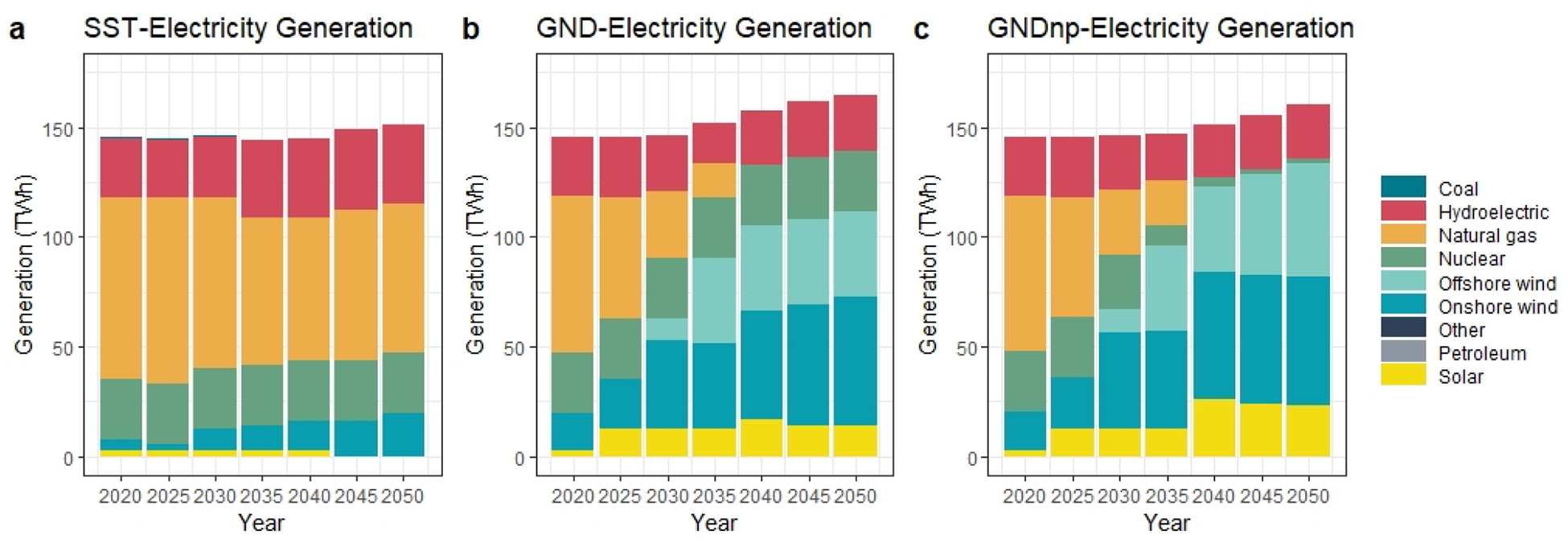
New York State power sector electricity generation (TWh) in (**a**) STEADY STATE (SST) scenario, (**b**) in GREEN NEW DEAL (GND) scenario, (**c**) GREEN NEW DEAL with the nuclear phase-out scenario (GNDnp) (Not that distributed solar is not included in the analysis).

Different scenarios result in different electricity generation mix choices. The implications of the assumptions in the reference case and Green New Deal scenarios can be observed through changes in the generation. Figure 5 shows that in the SST, within renewable sources, the highest utilization belongs to hydropower, whereas the contribution of other renewable sources especially solar remains low in the long run compared to other scenarios. Besides hydropower, nuclear power is also popular as a cost-effective source of electricity generation. The use of natural gas is dominating the primary energy supply for electricity generation in the SST scenario, whereas in both alternative policy scenarios, we see a decline in natural gas consumption due to the renewable portfolio share target. In GND and GNDnp scenarios, electricity generation from natural gas is expected to hit zero by 2040, enforced by the “zero emission” target. Figure 5 also indicates the considerable reduction in natural gas use in GND and GNDnp even in the 2025–2040 period compared to the BAU.

Within non-fossil-based energy resources, offshore wind fulfills the need for electricity resulted from the capacity reduction of nuclear power plants in GNDnp. Hence, we observe a 33% increase in the generation from offshore wind in GNDnp compared to the GND scenario. Regarding solar, 10 TWh additional generation is expected in 2050 in GNDnp.

**Figure 6.**
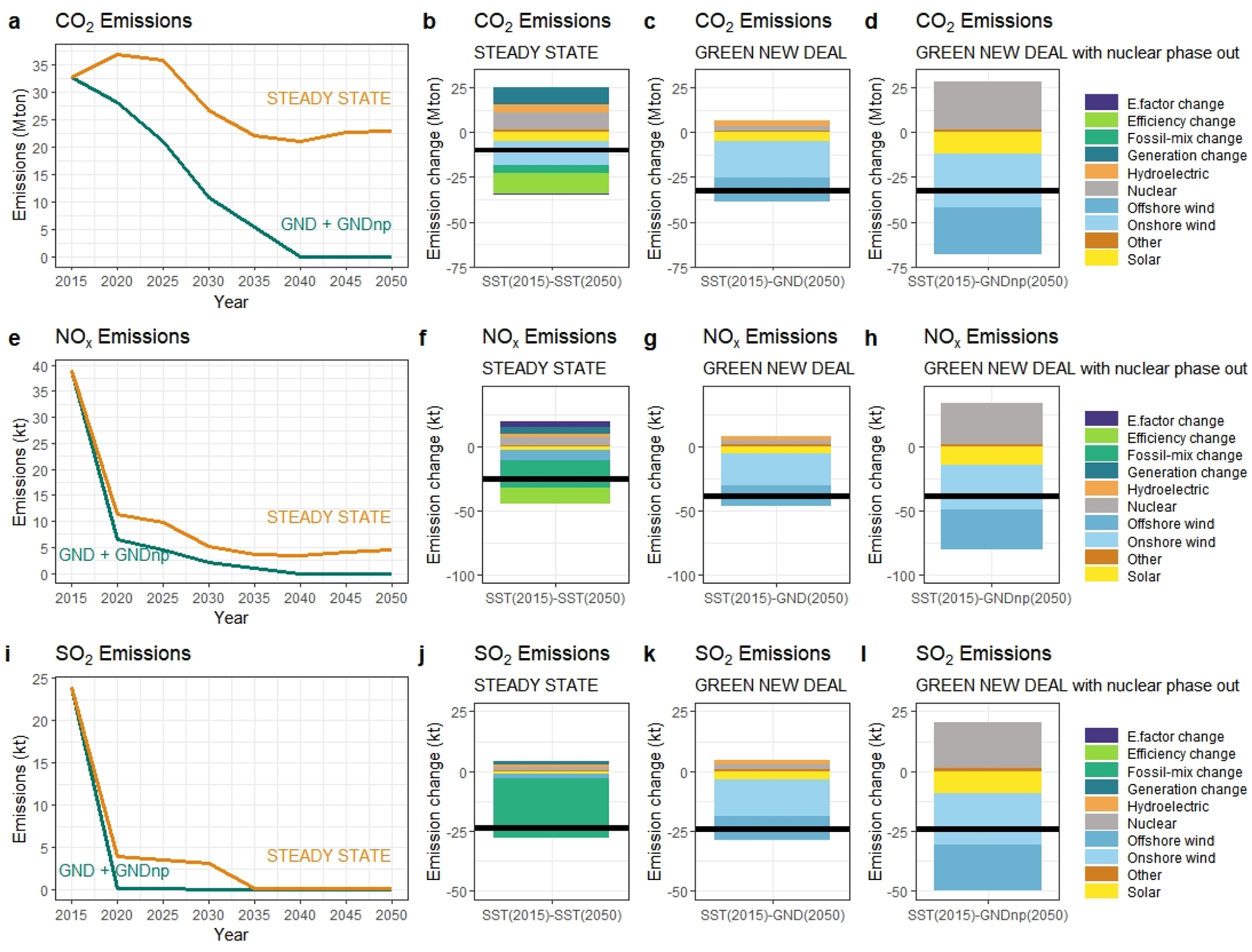
Results of LMDI decomposition analysis of the New York State power sector from 2015 through 2050 (Black line that intersects y-axis represent net emission change).

The impact of the associated technological choices and the accompanying supply mix of the scenarios on CO_2_, NO_x_, and SO_2_ emission levels are depicted in Figure 6. In the SST scenario, New York State’s power sector emissions are expected to reach 22.9 Mton CO_2_, whereas, in the GND and GNDnp scenarios, the CO_2_ emission levels follow a decreasing trend since we impose a constraint that ensures carbon neutrality by 2040. In SST, although the absolute value of the electricity generation from hydropower and nuclear does not fluctuate significantly, the share of electricity generated from those sources is expected to drop (for hydropower, from 19% to 14% and for nuclear generation from 32% to 22%). Hence, we observe an increase in emissions as given in Figure 6b, f, and j. The emissions have been expressed as the respective factor contributions to the total emissions. For SST, fossil fuel mix change and the electricity generated from wind provides the highest contributions to emissions reduction.

On the other hand, according to the MARKAL results, carbon neutrality can be achieved with a fuel and technology mix that significantly differs in reference to SST. The closure of nuclear power is expected to put an additional burden on the power sector to reach carbon neutrality. Hence, the shutting down of nuclear plants requires the replacement of this capacity by alternative resource options. This study demonstrates that onshore wind and offshore wind are steadily replacing nuclear also with the contribution of solar throughout the analysis period. Surprisingly, this study also depicts that, there exists no significant change in the electricity generated from hydropower regardless of the available capacity of nuclear power plants. The contributions of onshore and offshore wind are expected to be 20.41 Mton CO_2_ and 13.61 Mton CO_2_ in GND. However, when the nuclear capacity is reduced because of the phase-out actions, the total amount of abatement will be 29.47 Mton CO_2_ and 26.11 Mton CO_2_ for onshore and offshore wind respectively.

Consistent with this CO_2_ analysis, we also observe similar patterns in NO_x_ and SO_2_ emissions under GND and GNDnp scenarios. The reduction in the electricity generated by nuclear power is expected to result in an additional 28.5 kt NO_x_ and 17.6 kt SO_2_. As nuclear power is expected to be partially replaced by wind and solar, our study points out that onshore wind will abate 44% and 28% more of NO_x_ and SO_2_ to dampen the increase due to the phase-out as given in Figure 4. In comparison, offshore wind is expected to double its contribution to NO_x_ abatement, which is equivalent to 31.7 kt. Similarly, for SO_2_ this amount is expected to be 19.1 kt.

The MARKAL model also reports the discounted total system cost, which covers the endogenously generated fuel and electricity prices, processing and transmission costs, and the technology level investment, operation, and maintenance costs. To provide a better understanding of how emission reduction goals affect the power sector, the total discounted system cost is used to compare the overall price of New York City’s power sector between different scenarios. Costs are reported in 2005 $US Million and discounted by taking the inflation rate (5%) into account. The cost results show that there is great variability between SST and zero-emission scenarios. SST is expected to be $189,599, whereas GND is projected to reach $252,848. This study also finds that nuclear phase out does not lead to a significant increase in total system cost. GNDnp is estimated to cost around $262,080 when considering the New York State expenses solely.

## 4. Discussion

We find that carbon emissions in New York State’s power sector are expected to decrease by 67% between 1990 and 2050 under the SST scenario, with an absolute reduction of 46 Mton CO_2_. According to the projections, CO_2_ emission mitigation goals also have significant air emissions co-benefits under both GND and GNDnp scenarios.

The fossil fuel mix changes play a crucial role in emission mitigation while switching to non-fossil-based energy sources and generation efficiency are also dominant factors. However, the magnitude of those factors’ impacts differs for each period and each emission type. The LMDI decomposition results indicate that the transformation of the fossil fuel mix is the strongest driver regardless of the emission type, especially from 1990 to 2018. However, our analysis reveals that, as a future direction, renewables hold a higher potential than fossil-fuels in emission mitigation, as shown in Figure 6.

For the analysis of 2018–2050, we calculate electricity generation and related emissions data by deploying the MARKAL model. The MARKAL model results are included in the LMDI model to analyze the power sector from 2018 to 2050. We shed light on possible emission outcomes of the state’s electricity generation transformation. To accentuate the potential benefits of the net-zero emission target, we also built the SST scenario. For that purpose, the SST scenario represents the business as usual future of the New York State’s power sector.

In the SST scenario, as a consequence of the MARKAL model structure, results favor low-cost energy sources and technologies unless constraints are limiting their usage. Hence, we observe the cheapest electricity generation mix and resulting emissions under the SST scenario as given in Table 2. Although renewable energy options show progress in installation costs, they are still not as cost-effective as the fossil-based counterpart. Hence, their contribution is limited in SST. Besides the SST scenario, we also demonstrate two alternative future scenarios to show the impact of nuclear power and renewable energy alternatives under emission mitigation constraints. Scenario analysis demonstrates an approximately 40% increase in total system cost in alternative future scenarios in reference to the SST scenario.

The increasing electricity generation efficiency restrained the CO_2_ emissions in New York State power sector, especially between 2000 and 2010. Emission reduction due to Cu equals 8.1 Mton CO_2_ during this period, accounting for more than 20% of the aggregate deviation. Considering the existing generation fleet and the New York State’s Clean Energy Standard plan, our findings illustrate that efficiency increase is not expected to be a significant driver of emissions under GND and GNDnp scenarios. Suppose the targeted renewable penetration is not attained. In that case, our results indicate that efficiency change will possibly be a significant driver of carbon and NO_x_ abatement, as we observe under the SST scenario.

Regarding criteria pollutant emissions, although renewables are the central component of New York State’s Clean Energy Standard plan, they do not have as a significant effect on NO_x_ and SO_2_ mitigation from 1990 to 2018. For those emissions, technical efficiency gains and installed pollution controls were more influential than other factors included in the decomposition analysis. The effect of the change in fossil fuel share to non-fossil-based sources (S_p_ and N_p_) did not impact air pollutions as substantially as air regulations and the change in the fossil fuel mix from 1990 to 2018. The implementation of CAAA resulted in a rapid reduction of NO_x_ and SO_2_ emissions until 2005. The biggest trigger of SO_2_ emission reduction was the shift from coal power plants to natural gas-fired electricity generation units.

Under GND and GNDnp scenarios, we see a similar pattern in contributing factors. Because of the extreme penetration of renewable sources, C_u_, N_u_, and S_u_ turn out to be essential emission drivers. Hydropower, with its low unit production cost, competes with fossil-based resources already. Since it generates electricity close to its potential, no additional significant capacity addition is observed. Hence, hydropower’s contribution to emissions mitigation is expected to be limited even under the nuclear phase-out. Solar, onshore wind and offshore wind alternatives exert sufficient downward pressure on natural gas, and they provide a significant contribution to the emission mitigation actions.

Given the results, one of the most striking findings of the model is that results do not demonstrate significant changes in the total system cost. The deviation in the total system cost appears to be limited to 4%. The evidence presented supports that onshore and offshore wind are very effective generation options in achieving carbon neutrality in the long run. In summary, when the overall results of these three scenarios are considered, should nuclear power plants phased out, the electricity generation deficit will be balanced by the offshore wind technology under extreme decarbonization targets.

The study that we present here has some limitations. It assumes that NYS’s electricity demand follows a similar pattern with EPAUS9r’s North-East Region electricity generation from 2018 to 2050 under a business as usual scenario. We assume that all energy-consuming sectors will follow existing trends without any specific emission reduction targets (no extreme electrification is expected on the demand side). Accordingly, the impact of extreme-decarbonization of end-use energy services on electricity demand is not captured.

## 5. Conclusions

Electricity generation is one of the significant contributors to anthropogenic CO_2_ emissions. As a response, local governments, including the New York State, are setting targets to limit CO_2_ emissions to avert their negative impact on the environment. These mitigation actions also have air quality implications. The purpose is to identify the drivers of emissions considering the existing data, but we also aim to show the potential co-benefits of the State’s CO_2_ mitigation target. Hence the period of the study is selected as 1990–2050. We applied two-phased LMDI to identify the underlying factors on the deviation of CO_2_, NO_x_, and SO_2_ emissions from New York State’s power sector. The analysis between 1990–2018 relies on the reported data, whereas, for the rest of the analysis period, MARKAL model results are used in the decomposition analysis. We try to illustrate how NYS’s electricity generation is realized under alternative scenarios and how emissions and system costs change concerning those scenarios. While this analysis provides in-depth insights on each factor type (i.e., technology efficiency, fuel type, emission factors), we especially focus on renewable energy options and calculate their contributions to exhibit deeper insights about the emission change as an input for future emission mitigation actions.

The New York State’s total annual electricity generation level did not fluctuate drastically during the 1990–2018 period, yet power sector emissions declined significantly. The most important finding of this study is that no unique contributor can provide the highest benefit for CO_2_, NO_x_, and SO_2_ together for each period in the analysis. The reduced natural gas prices boost the considerable change in the electricity production portfolio. It provided a significant contribution in terms of a decline in emission level. Thus, approximately half of this reduction is due to C_m_, the fossil fuel mix change. The same observation is valid for SO_2_ emissions, especially between 2005 and 2018. However, for NO_x_, the technical transformation of electricity generation facilities is more important.

Even though significant long-term aggregate abatement potentials exist, short-term trends (especially after 2000) show impacts at different levels. In terms of NO_x_ emissions, N_m_ became ineffective and only covered 7% of NO_x_ reduction after 2005, although it was also an essential component between 1990 and 2005.

We conduct scenario analysis to explore each non-fossil-based energy resources’ contributions considering the Green New Deal target with and without nuclear phase-out. Our findings reveal that offshore wind and onshore wind appeared to be the critical factors of emission mitigation actions. Specifically, offshore wind plays an important role in the GND scenario with nuclear phase-out replacing retired nuclear power plant capacity cost-effectively to compensate for the additional need to reduce CO_2_ emissions. In both policy scenarios, the State can still achieve its CO_2_ emission mitigation target with significant air emission co-benefits. Although solar has the potential to reduce CO_2_ emissions, compared with other renewable options, its contribution is limited.

With an increase in electricity demand in end-use sectors such as buildings and transportation, the power sector has been the primary determinant of broader air and GHG emissions. As countries, regions, states implement either broader or sectoral emission reduction targets for air and GHG emissions, more attention should be given to the power sector’s direct and indirect influence on emissions. Our results imply that the magnitude of fossil fuel mix change diminishes over time because of the power sector’s switch to cleaner fuels. We show that, however, there is still room for improvement in reducing CO_2_, NO_x_, and SO_2_ emissions. Within alternative mitigation options, advancements in technological efficiency and implementation of air regulations offer the most significant potential to reduce emissions. Although the study is built upon New York State’s data, the methodology can be applied, and insights can be extended to other locations.

## Figures and Tables

**Table 1.** Scenario assumptions for New York State.

Scenario	Steady State	Green New Deal	Green New Deal + Nuclear Phaseout
**Coal**	No additional constraints	Generation from coal is not allowed in 2020 and thereafter	Generation from coal is not allowed in 2020 and thereafter
**Natural gas, hydro, onshore wind**	No additional constraints	No additional constraints	No additional constraints
**Nuclear**	No constraints on nuclear power plants	Indian Point power plant is shut down. No constraint on future investments	Indian Point power plant is shut down. Future investment is not allowed in 2020 and thereafter
**Offshore wind**	No constraints on offshore wind	9000 megawatts by 2035	9000 megawatts by 2035
**Electricity transmission and distribution**	Maximum annual energy losses due to distribution and transmission are 4.56% and 6.5% respectively	Maximum annual energy losses due to distribution and transmission are 4.56% and 6.5% respectively	Maximum annual energy losses due to distribution and transmission are 4.56% and 6.5% respectively
**Emissions**	No constraints on emissions	100% clean power by 2040	100% clean power by 2040
**Renewables**	No constraints on renewables	At least 70% of electricity generation by renewable power by 2030	At least 70% of electricity generation by renewable power by 2030

**Table 2.** Scenario analysis results from 2015 to 2050.

	Steady State	Green New Deal	Green New Deal + Nuclear Phaseout
**Aggregate CO**_2_ **emissions**	220.5 Mton	97.8 Mton	99.0 Mton
**Aggregate NO**_x_ **emissions**	80.9 kt	53.1 kt	53.1 kt
**Aggregate SO**_2_ **emissions**	35.0 kt	23.9 kt	23.9 kt
**Share of fossil-based electricity generation**	50%	14.2%	16.7%
**Share of nuclear-based electricity generation**	18.9%	15.9%	9.3%
**Total System Cost**	189,599 (Million $)	252,848 (Million $)	262,080 (Million $)

## Abbreviation

t: Time
T: Targeted time
i: The type of fuel
j: The type of non-fossil fuel
l: All non-fossil fuel-based sources
C^t^: Total CO_2_ emissions from electricity generation in year t
G: The total electricity generation
Q: The sum of the electricity generated from all fossil fuels
Q_i_: The total electricity generation from fossil fuel i
F_i_: Energy input from fossil fuel i
C_i_: Total CO_2_ emissions from fuel i
p = Q/G: The percentage of electricity generated from fossil fuels
m_i_ = Q_i_/Q: The percentage of electricity generated from fossil fuel i
u_i_ = F_i_/Q_i_: The ratio of energy input to electricity generated from fossil fuel i
e_i_ = C_i_/F_i_: Emission factor for fossil fuel i
ΔC_tot_: A total change of CO_2_ emissions
ΔC_G_: The change in total electricity generation effect on CO_2_ level
ΔC_p_: The effect of the change in the share of fossil fuels to non-fossil-based sources on CO_2_ level
ΔC_pj_: The effect of the change in the share of non- fossil fuel j on CO_2_ level
ΔC_m_: The effect of changes in fossil fuel mix on CO_2_ level
ΔC_u_: The effect of generation efficiency on CO_2_ level
ΔD_tot_: The change in the ratio
ΔN_tot_: A total change of NO_x_ emissions
ΔC_e_: The effect of the change in fuel emission factors on CO_2_ level
ΔN_G_: The change in total electricity generation effect on NO_x_ level
ΔN_p_: The effect of the change in the share of fossil fuels to non-fossil-based sources on NO_x_ level
${\Delta \text{N}}_{{\text{P}}_{\text{j}}}$: The effect of the change in the share of non- fossil fuel j on NO_x_ level
ΔN_m_: The effect of changes in fossil fuel mix on NO_x_ level
ΔN_u_: The effect of generation efficiency on NO_x_ level
ΔN_e_: The effect of the change in fuel emission factors on NO_x_ level
ΔS_tot_: A total change of SO_2_ emissions
ΔS_G_: The change in total electricity generation effect on SO_2_ level
ΔS_p_: The effect of the change in the share of fossil fuels to non-fossil-based sources on SO_2_ level
${\Delta \text{S}}_{{\text{P}}_{\text{j}}}$: The effect of the change in the share of non- fossil fuel j on SO_2_ level
ΔS_m_: The effect of changes in fossil fuel mix on SO_2_ level
ΔS_u_: The effect of generation efficiency on SO_2_ level
ΔS_e_: The effect of the change in fuel emission factors on SO_2_ level
